# The role of the diaphragm in prediction of respiratory function in the immediate postoperative period in lung cancer patients using a machine learning model

**DOI:** 10.1186/s12957-023-03278-1

**Published:** 2023-12-22

**Authors:** Radomir Vesovic, Milan Milosavljevic, Marija Punt, Jelica Radomirovic, Slavisa Bascarevic, Milan Savic, Vladimir Milenkovic, Marko Popovic, Maja Ercegovac

**Affiliations:** 1 Faculty of Medicine, University of Belgrade, Dr Subotica 8, 11000 Belgrade, Serbia; 2 Clinic for Thoracic Surgery, University Clinical Center of Serbia, Koste Todorovica 26, 11000 Belgrade, Serbia; 3 Vlatacom Institute of High Technology, Bulevar Milutina Milankovica 5, 11000 Belgrade, Serbia; 4 School of Electrical Engineering, University of Belgrade, Bulevar Kralja Aleksandra 73, 11000 Belgrade, Serbia

**Keywords:** Diaphragm, Respiratory function, Machine learning, Lung cancer, Prediction

## Abstract

**Background:**

The prediction of postoperative respiratory function is necessary in identifying patients that are at greater risk of complications. There are not enough studies on the effect of the diaphragm on postoperative respiratory function prediction in lung cancer surgical patients. The objective of this study is to estimate the precision of machine learning methods in the prediction of respiratory function in the immediate postoperative period and how diaphragm function contributes to that prediction.

**Materials and methods:**

Our prospective study included 79 patients who underwent lung cancer surgery. Diaphragm function was estimated by its mobility measured both ultrasonographically and radiographically and by noninvasive muscle strength tests. We present a new machine learning multilayer regression metamodel, which predicts FEV1 for each patient based on preoperative measurements.

**Results:**

The proposed regression models are specifically trained to predict FEV1 in the immediate postoperative period and were proved to be highly accurate (mean absolute error in the range from 8 to 11%). Predictive models based on resected segments give two to three times less precise results. Measured FEV1 was 44.68% ± 14.07%, 50.95% ± 15.80%, and 58.0%1 ± 14.78%, and predicted postoperative (ppo) FEV1 was 43.85% ± 8.80%, 50.62% ± 9.28%, and 57.85% ± 10.58% on the first, fourth, and seventh day, respectively. By interpreting the obtained model, the diaphragm contributes to ppoFEV1 13.62% on the first day, 10.52% on the fourth, and 9.06% on the seventh day.

**Conclusion:**

The machine learning metamodel gives more accurate predictions of postoperative lung function than traditional calculations. The diaphragm plays a notable role in the postoperative FEV1 prediction.

## Introduction

Preoperative assessment of postoperative respiratory function is routinely performed in patients with moderate to severe chronic obstructive pulmonary disease (COPD) who are being prepared for lung cancer surgery [1]. The interaction between COPD and lung cancer is common among patients considering that smoking is a great risk factor for both lung cancer and COPD, and that COPD is an important independent risk factor for lung cancer [2]. Postoperatively, COPD patients have lower survival outcomes than non-COPD patients [3]. The assessment of postoperative respiratory function is recommended in identifying patients at greater risk of complications [1]. Predicted postoperative forced expiratory volume in 1 s (ppoFEV1) is most commonly used in postoperative respiratory function and risk factor estimation. PpoFEV1% calculation methods based on removed segments, subsegments, or functional segments are still widely used given their simple application in clinical practice [1, 4–6].

However, there is often a certain difference between predicted and postoperatively measured respiratory parameters. It has been shown that there is a strong correlation between ppoFEV1% and measured FEV1% in the 3- to 6-month period after surgery, while in the immediate postoperative period, when most of the complications arise, the correlation is weak. In the first postoperative days, the difference between predicted and measured FEV1% can reach 30% [7].

The role of the diaphragm in postoperative respiratory function prediction and in postoperative respiratory complications in lung cancer surgical patients has not been significantly studied in literature [8]. There are indirect data, from meta-analysis, about the potential significance of inspiratory muscles strength, which is mostly diaphragm strength, in reducing postoperative pulmonary complications [9]. The potential significance of the diaphragm is also shown by the fact that preoperative diaphragm dysfunction contributes to respiratory complications in cardiac surgery patients, and that it was associated with prolonged mechanical ventilation after lung transplantation [10, 11].

Recently, machine learning models have been used in clinical practice to estimate outcomes and predict postoperative lung function and risk of complications after lung surgery [12–14]. Previous studies have shown that machine learning models potentially have higher accuracy than conventional statistical methods [12].

The aim of this study is to assess the precision of machine learning methods in the prediction of postoperative lung function in the immediate period after surgery and how mobility and strength of the diaphragm contribute to the aforementioned prediction.

## Materials and methods

### Study design and participants

The prospective cohort study included 79 patients. They had resection performed for primary non-small cell lung cancer at the Clinic for Thoracic Surgery of the University Clinical Center of Serbia from January 2015 to October 2016. Lung resection was done by muscle-sparing thoracotomy with extension towards complete posterolateral thoracotomy when it was necessary.

Inclusion criteria in the study were as follows: full cooperation of a patient while measuring diaphragm movements, proven primary lung cancer and complete assessment of functional status, and overall cardiorespiratory risk.

Exclusion criteria were as follows: poor cooperation of a patient during the required measurements, prior surgery in abdomen and thorax, definitive histopathological findings that indicate another disease, existence of neuromuscular and skeletal diseases, resection of chest wall deemed to be necessary, the presence of massive adhesions observed radiographically preoperatively, and the presence of a ventral hernia of abdominal wall.

### Measurements of respiratory function and respiratory muscles strength

Preoperative lung function was measured upon admission to the hospital and classified according to the GOLD criteria [15]. Postoperative respiratory function was measured on the first, the fourth, and the seventh day after surgery. Measurements were taken three times, out of which the highest result was used in the analysis.

Preoperative spirometry and body plethysmography were done with a pneumotachometer (MasterScreen Pneumo, Viasys Healthcare, Germany), while postoperative spirometry was done with a SpiroPRO portable pneumotachometer (Viasys, Germany).

Tests of respiratory muscles strength, maximal inspiratory pressure (PImax), maximal expiratory pressure (PEmax), and sniff nasal inspiratory pressure (SNIP) were completed with MicroRPM (Care Fusion, San Diego, CA, USA) according to ATS/ERS guidelines [16].

Postoperative measurements were taken under the maximal analgesia (NSAID and tramadol-chloride), and analgetics were administered in equal intervals during the first 48 to 72 h and after that upon the patient’s request.

These patients had both hemidiaphragms movements measured radiographically and ultrasonographically preoperatively along with respiratory function.

### Radiographic measurement

Preoperative radiography was done in the radiology department with a patient in the upright position.

Using standard chest radiography, the distance between the inferior margin of the second rib posteriorly and a horizontal tangent line to diaphragm dome was measured in maximum inspirium (distance a) and maximum expirium (distance b). Preoperative diaphragm movement amplitude (A) was calculated by subtracting the aforementioned distance in expirium (b) from the same distance measured in inspirium (a): A = a-b [8].

The measurement was performed separately for the right and left hemidiaphragms.

### Ultrasonographic measurement

Ultrasound measurement of diaphragm movement was done using the Nemio XG MK1 apparatus (Toshiba, Japan).

Once a patient is in a supine, 45° semi-recumbent position, a 3.75-MHz convex transducer was symmetrically placed subcostally between the mid-clavicular and mid-axillary line to obtain a sagittal plane of the hemidiaphragm during every respiratory phase. Upon identification of the right and left hemidiaphragm, two-dimensional (2D) scans were taken, by using a real-time gray scale technology in the sagittal plane that included maximal renal bipolar length. Using 2D images, the hemidiaphragm location was determined relative to the renal pelvis. The point was marked where the hemidiaphragm was observed during craniocaudal excursion at the end of deep expiration. The other point was recorded at the end of the maximum inspiration with the diaphragm lying at the same depth from the transducer on the ultrasound scan. The distance between these points represents the diaphragm mobility measured by ultrasound technique [8].

These measurements were taken three times, for each hemidiaphragm, out of which the best value was used in the analysis.

### Estimation of respiratory function

Predicted postoperative FEV1% (ppoFEV1%) was calculated as follows:

- By using the formula developed by Nakahara (N) and associates [5]:
  1 $$\text{PpoFEV}1\%=\lbrack1-(\text{n}-\text{a})/(42-\text{a})\rbrack\times\mathrm{preoperative}\;\mathrm{FEV}1\%$$
  where (n) is the number of resected subsegments in the lobe, that is, 6, 4, and 12 for right upper, middle, and lower lobe and 10 for left upper and lower lobe, while (a) is the number of subsegments obstructed by a tumor.
- By using the Juhl-Frost (JF) formula [4]:
  2 $$\text{PpoFEV}1\%=\mathrm{preoperative}\;\mathrm{FEV}1\%\times(1-0.0526\times\mathrm{number}\;\mathrm{of}\;\mathrm{resected}\;\mathrm{lung}\;\mathrm{segments})$$
  which means for upper right lobe 3 segments, middle 2 segments, left upper lobe 4 segments, and lower lobes 5 segments.
- ERS/ESTSa guidelines formula based on removal on functional segments (FS) [1]:
  3 $$\text{PpoFEV}1\%=\mathrm{preoperative}\;\mathrm{FEV}1\%\times(1-\text{a}/\text{b})$$
  where (a) is the number of obstructed segments that are to be resected and (b) is the total number of unobstructed segments.

In pain level assessment, the visual analogue scale was used.

The study was conducted in accordance with the World Medical Association Declaration of Helsinki and in accordance with the relevant guidelines and regulations. This study protocol was reviewed and approved by the Ethics Committee of the University of Belgrade, Faculty of Medicine, approval number 29/XII-10. All subjects gave written informed consent before participation. The authors received no financial support for this research.

### Model training and analysis

A dataset ($${X}_{i},{y}_{i}),$$ $$i=\mathrm{1,2},\dots ,N$$ where $$N$$ is the number of patients who underwent surgery is available. The vector $${X}_{i}$$ represents the M-dimensional vector of features, measured preoperatively, while the variable $${y}_{i}$$ is the measured value of the postoperative FEV1% which is standard indicator of respiratory function.

Given that we have the set ($${X}_{i},{y}_{i}),$$ $$i=\mathrm{1,2},\dots ,N$$, we will create the best possible predictive model within the machine learning methodology.

4 $${\widehat{y}}_{i}=F\left({X}_{i}\right)$$

Since $y$ is a continuous variable, (4) is actually a general nonlinear regression model. The first problem to be solved is identification of mapping $F\left({X}_{i}\right)$ that gives the highest possible accuracy, measured by the criterion of mean absolute error, MAE:

5 $$MAE=\sum_{i=1}^{N}\left|{\widehat{y}}_{i}-{y}_{i}\right|.$$

When the regression model (4) is sufficiently accurate, it is possible to estimate the contribution of each of the input variables of the model to the output prediction of FEV1% [17]. It is interesting to note that this issue has gained actuality within the newly emerging field of study called explainable artificial intelligence [18].

Postoperative FEV1% was measured on the first, the fourth, and the seventh day after surgery. These variables were selected for output variable $y$ in this model (4). This means that a separate regression model is trained for each output variable, so a total of three models. The first 25 characteristics from Table 1 are used as input features. The model selection and construction were executed using the scikit-learn library [19] in the Python programming language. For data analysis, we used the NumPy and Pandas libraries to gain insights into the patients’ characteristics [20, 21].

**Table 1:** Patient characteristics

No	Characteristic		Values (mean ± std. or frequency (%))
**1**	**Age**		60.24 ± 7.31
**2**	**Type of operation on the right lung**	**Operation is not on the right lung**	32 (40.51%)
		**Right upper lobectomy**	18 (22.78%)
		**Right bottom lobectomy**	14 (17.72%)
		**Right middle lobe lobectomy**	3 (3.80%)
		**Bilobectomy superior**	2 (2.53%)
		**Bilobectomy inferior**	3 (3.80%)
		**Right pneumonectomy**	7 (8.86%)
**3**	**Type of operation on the left lung**	**Operation is not on the left lung**	47 (59.49%)
		**Left upper lobectomy**	15 (18.99%)
		**Left bottom lobectomy**	9 (11.39%)
		**Left pneumonectomy**	8 (10.13%)
**4**	**BMI (**$\frac{{\varvec{k}}{\varvec{g}}}{{{\varvec{m}}}^{2}}$**)**	**Underweight**	1 (1.27%)
		**Normal weight**	33 (41.77%)
		**Overweight**	29 (36.71%)
		**Obesity**	16 (20.25%)
**5**	**Type of respiratory function**	**Normal respiratory function**	33 (41.77%)
		**Mild (stage I)**	39(49.37%)
		**Moderate (stage II)**	6 (7.59%)
		**Severe (stage III)**	1 (1.27%)
**6**	**COPD index = (FEV1% + FEV1%/FVC)/100**		1.66 ± 0.22
**7**	**Preoperative FEV1%-preoperative forced expiratory volume in the first second %**		94.53 ± 15.63
**8**	**Preoperative VC %-preoperative vital capacity %**		109.23 ± 15.87
**9**	**Preoperative FVC %-preoperative forced vital capacity %**		107.86 ± 15.04
**10**	**Preoperative VCin %-preoperative vital capacity in inspiration %**		105.51 ± 14.20
**11**	**Preoperative FEV1%/FVC**		71.32 ± 9.41
**12**	**TLC %-total lung capacity %**		116.27 ± 14.41
**13**	**RV %-residual volume %**		137.13 ± 34.03
**14**	**FRC (ITGV) %-functional residual capacity %**		133.67 ± 27.32
**15**	**RV/TLC (% predicted)**		109.49 ± 20.25
**16**	**FRC (ITGV) % (% predicted)**		107.51 ± 18.53
**17**	**Mobility of the right hemidiaphragm measured radiographically**		4.16 ± 1.41
**18**	**Mobility of the left hemidiaphragm measured radiographically**		4.08 ± 1.39
**19**	**Mobility of the right hemidiaphragm measured by ultrasound**		68.25 ± 10.28
**20**	**Mobility of the left hemidiaphragm measured by ultrasound**		62.58 ± 11.10
**21**	**Preoperative PImax %-preoperative maximal inspiratory pressure %**		109.63 ± 35.76
**22**	**Preoperative PEmax %-preoperative maximal expiratory pressure %**		92.19 ± 18.04
**23**	**Preoperative snip %-preoperative** “**sniff**”** inspiratory pressure**		91.83 ± 23.26
**24**	**The number of functional segments removed by the operation**		3.41 ± 2.02
**25**	**The number of total functional segments in the lungs**		17.23 ± 1.07
**26**	**Sex**	**Male**	45 (56.96%)
		**Female**	34 (43.04%)
**27**	**Cancer stage**	**Ia**	14 (17.72%)
		**Ib**	13 (16.45%)
		**IIa**	26 (32.91%)
		**IIb**	10 (12.66%)
		**IIIa**	15 (18.99%)
		**IIIb**	1 (1.27%)
**28**	**Pain after operation**	**1st day**	29.41 ± 10.86
**29**		**4th day**	18.85 ± 10.46
**30**		**7th day**	11.70 ± 8.02
**31**	**Measured FEV1% after operation**	**1st day**	44.68 ± 14.07
**32**		**4th day**	50.95 ± 15.80
**33**		**7th day**	58.01 ± 14.78

Continuous variables are represented as mean value ± standard deviation, and categorical variables are represented as frequency and relative frequency

In order to measure the accuracy of our model, we will compare the obtained regression predictive models with existing methods of calculating ppoFEV1% based on preoperative measurements. We limited ourselves to three basic segment counting methods easily available in clinical practice given by (1), (2), and (3), respectively.

Finding the best model was divided into two phases. In the first phase of research, we examined the error of regression models based on individual basic regression algorithms. Table 2 shows the MAE of basic regression models with the default (recommended) hyperparameters for predicting postoperative FEV1% on the seventh day after surgery. Error was calculated by fivefold cross-validation. A lower value represents a better result. The default model hyperparameters are chosen according to the documentation of the used scikit-learn library [22, 23].

**Table 2:** Mean value and standard deviation of MAEs models for predicting postoperative FEV1% on the seventh day after surgery, obtained by fivefold cross-validation

No	Model	Values (mean ± std. or frequency (%))
1	**Extra tree**	8.64 ± 1.43
2	**Random forest**	9.05 ± 1.47
3	**SVM linear**	9.05 ± 2.06
4	**Lasso**	8.05 ± 2.01
5	**XGBoost**	10.09 ± 2.37
6	**Ridge regression**	9.98 ± 1.54
7	**KNN**	9.63 ± 1.69
8	**MLP1 (7, 3, 2)**	12.23 ± 1.36
9	**SVM.RBF**	11.38 ± 1.51
10	**MLP2 (3, 2)**	9.85 ± 2.00
11	**LightGBM**	8.90 ± 1.02
12	**AdaBoost**	9.45 ± 1.35

**Fig. 1:**
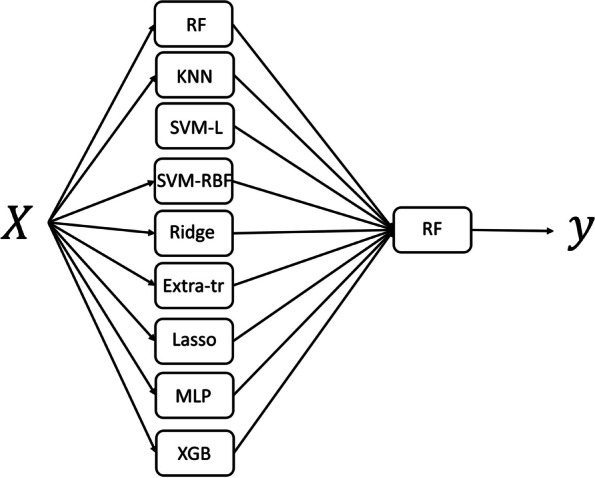
The architecture of the regression metamodel of depth 2

In the second phase of the research, regression metamodels were examined. By metamodels, we mean the combination of individual base models in the form of layered structures of a certain depth. The model architecture is shown in Fig. 1. It consists of a combination of previously described basic regression models in two layers, so depth of this metamodel is 2. This step proved to be justified, since the metamodel MAE is lower than that of each individual model.

The MAE of our metamodel is 7.98% ± 1.51%. This metamodel was also used in prediction of remaining output variables.

### Feature importance

For a trained model, it is significant to understand the importance of each feature on the model’s accuracy. One of the most developed techniques, which successfully answers this question, is the method based on calculation of the so-called SHAP (SHapley Additive explanations) values for each feature, first introduced in the work of Lundberg and Lee [17].

Benefits of this method are as follows:

- SHAP values can be calculated for a wide class of models, either by exact or approximate methods.
- Global interpretability, which consists in the fact that aggregated SHAP values show how much each feature (predictor) affects output variable (prediction), either positively or negatively

The procedure for calculating SHAP values for a given model consists of the following steps:

1. Form a cross-validation structure of given data.
2. Train the model on the current fold and calculate SHAP values of all features on corresponding test fold.
3. At the end of cross-validation, find mean value for SHAP values of each feature.
4. In order to obtain the cumulative effect of every feature associated with the diaphragm, the SHAP values of those features are summed up (mobility of left and right diaphragm measured radiographically, mobility of left and right diaphragm measured by ultrasound, preoperative PImax%, preoperative Snip%). This step is justified by the additivity of SHAP values.

## Results

Patient attributes are shown in Table 1. Most patients were male, 45 (56.96%), while 34 (43.04%) of them were female. Age group was 60.24 ± 7.31. Sixteen (20.25%) patients had a BMI greater than 30. The majority of the patients, 59 (74.68%), had a lobectomy performed, while 15 (18.99%) patients underwent pneumonectomy, and 5 (6.33%) had bilobectomy. Most of them, 63 (79.74%), were in cancer stages I and II, while 15 (18.99%) were in stage IIIa, and only one patient was in stage IIIb. According to GOLD criteria, 46 (58.22%) patients had COPD, with 39 of them (49.37%) having mild COPD and 6 (7.59%) moderate, and one patient had severe COPD.

Table 3 shows the MAE of the metamodel and traditional calculation methods for ppoFEV1%.

**Table 3:** MAE of the model for ppoFEV1%

MAE ppoFEV1%	1st day after surgery	4th day after surgery	7th day after surgery
Our metamodel	8.24 ± 0.93	10.56 ± 0.87	7.98 ± 1.51
Functional segments	31.30 ± 2.44	25.40 ± 2.51	18.86 ± 1.01
Juhl Frost	25.87 ± 1.79	20.03 ± 2.32	14.32 ± 1.64
Nakahara	29.57 ± 2.06	23.65 ± 2.40	17.11 ± 1.25

By comparing our machine learning model FEV1% predictions with predictions obtained by the JF model, as being the most precise out-of-segment counting methods, the following results are obtained. The machine learning model is inaccurateL 8.24% ± 0.93% on the first postop day, 10.56% ± 0.87% on the fourth, and 7.98% ± 1.51% on the seventh. JF method is inaccurate: 25.87% ± 1.79% on the first postop day, 20.03% ± 2.32% on the fourth, and 14.32% ± 1.64% on the seventh. Error of N and FS methods is shown in Table 3. Comparing the MAE of our metamodel with the most accurate JF method, we can notice that it is lower by two to three times (Table 3).

**Fig. 2:**
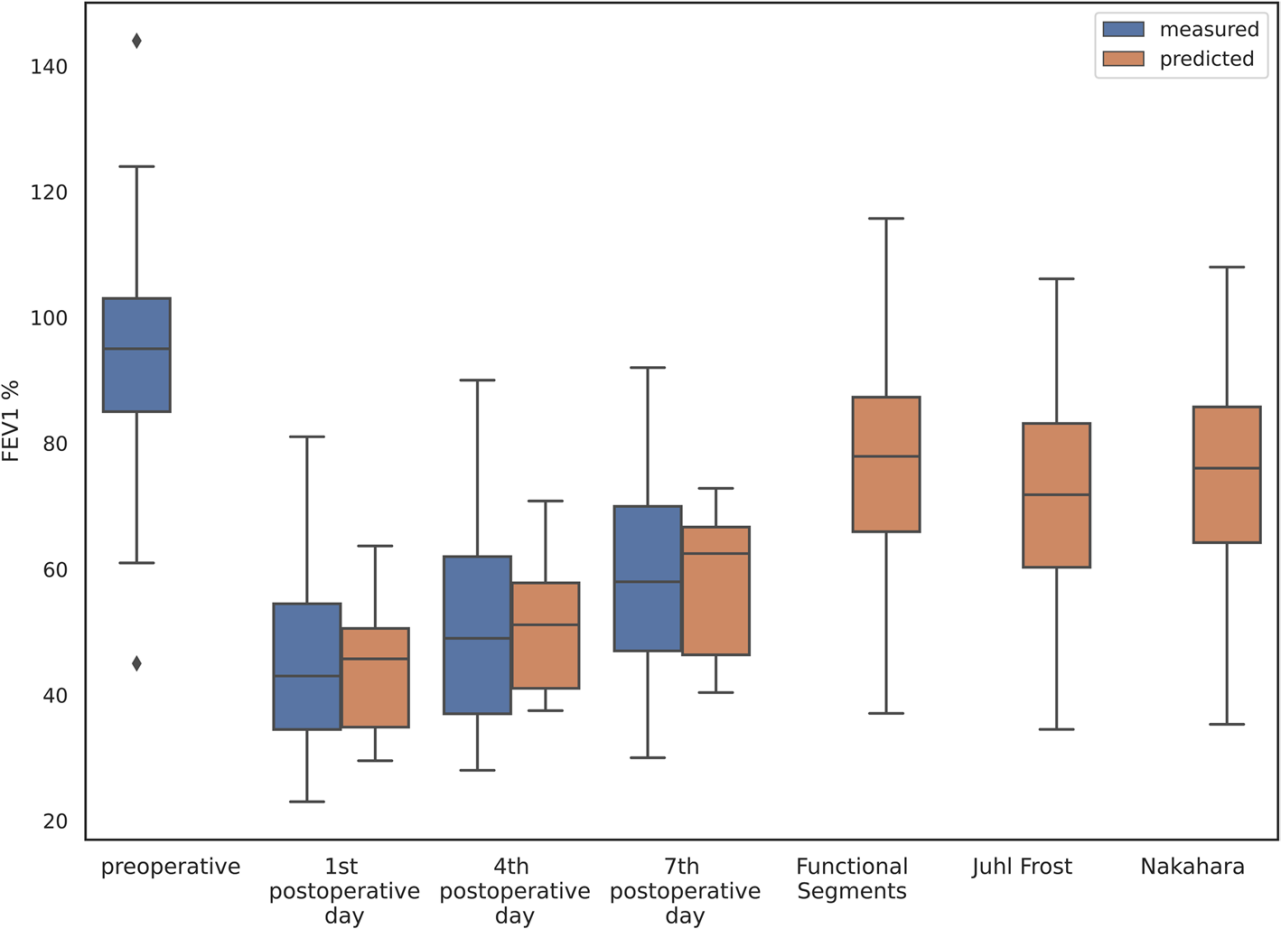
A boxplot of FEV1% representing the range of measured and predicted values

The machine learning model for predicting FEV1% on the first, the fourth, and the seventh postoperative day demonstrates almost identical mean values compared to the measured postoperative FEV1% on the equivalent days (Table 4, Fig. 2). The difference between the two is − 0.83% on the first day, − 0.33% on the fourth, and − 0.16% on the seventh. Discrepancies in standard deviations are indicated in the same Table 4.

**Table 4:** Measured and predicted values of FEV1%

	**Mean ± std**
FEV1% 1st day	44.68 ± 14.07
FEV1% 4th day	50.95 ± 15.80
FEV1% 7th day	58.01 ± 14.78
ppoFEV1% 1st day	43.85 ± 8.80
ppoFEV1% 4th day	50.62 ± 9.28
ppoFEV1% 7th day	57.85 ± 10.58
Functional segments	75.91 ± 17.40
Juhl Frost	69.91 ± 17.50
Nakahara	74.01 ± 17.21

We also used segment counting methods that are widely applied in clinical practice, in measuring the difference between the predicted and measured FEV1% in order to be able to compare them with the aforementioned results obtained by our machine learning model. The results are the following: on the first postoperative day, the difference between the predicted and actual FEV1% is 31.23% measured by FS method, 25.23% by JF, and 29.33% by N. On the fourth postop day, those differences are 24.96%, 18.96%, and 23.06%, while on the seventh postop day, they are 17.9%, 11.9%, and 16%, measured by FS, JF, and N methods, respectively. These results suggest that the FS method is the least precise, while the JF is the most precise. Going forward from the first to the seventh day, the FEV1% is recovering, and the measured values are approaching the predicted ones (Table 4, Fig. 2).

**Fig. 3:**
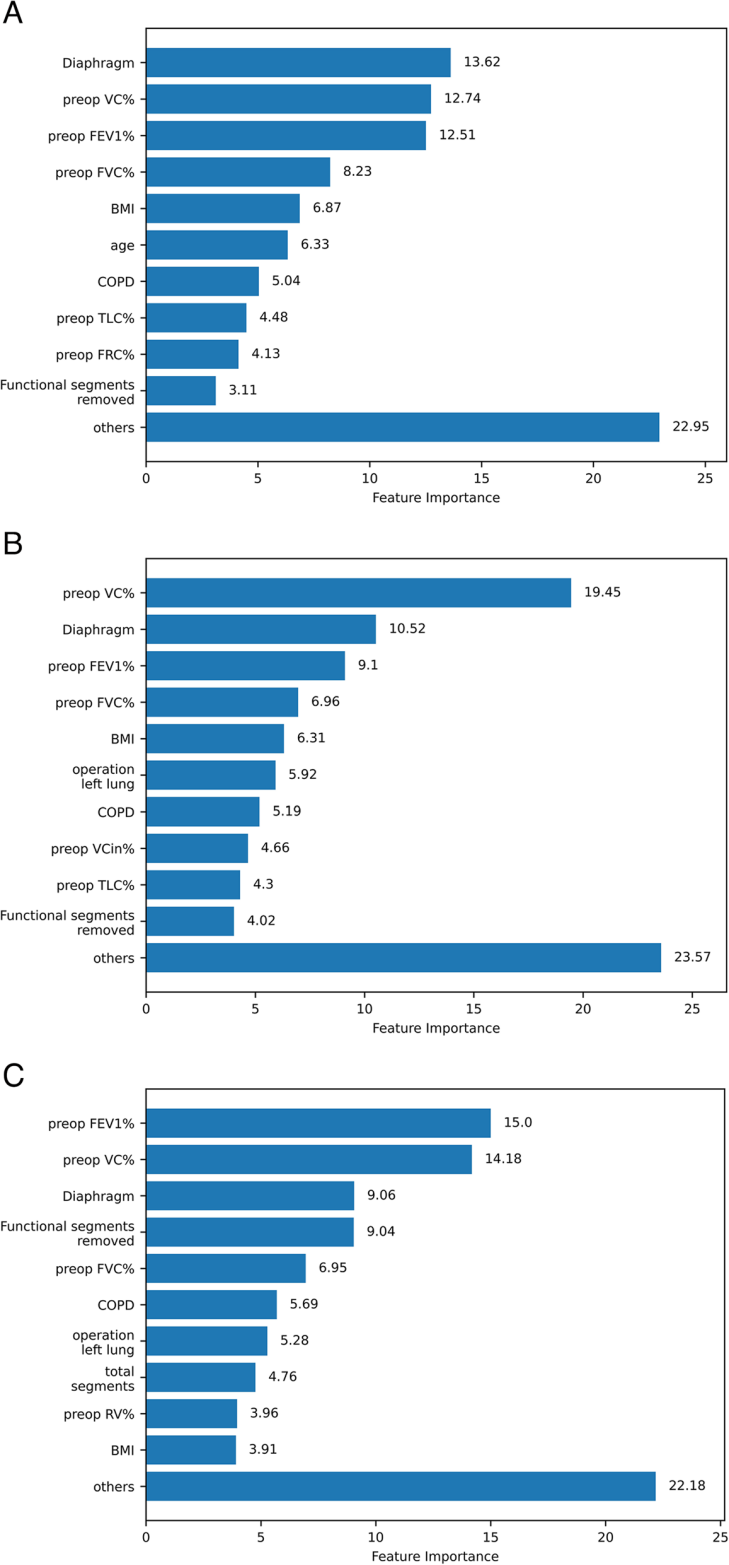
The top 10 most important features for the metamodel and the output variable ppoFEV1%. **a** The 1st, **b** the 4th, and **c** the 7th day after surgery, in descending order of significance calculated from the SHAP values

Following the steps described for calculating the SHAP values, we get the feature importance representing the impact of various preoperative factors on the ppoFEV1% (Fig. 3).

On the first postoperative day, the diaphragm has the largest impact in the FEV1% prediction, and its mobility has an influence of 9.80%. Combined with the effects of muscle strength tests, PImax 1.0%, and SNIP 2.82% (not shown in figures separately), the total influence of the diaphragm on ppoFEV1% is 13.62%. Other most important factors are spirometry parameters; see Fig. 3a.

On the fourth postoperative day, the role of the diaphragm in ppoFEV1% is slightly less significant, and the impact of diaphragm mobility is 6.53% PImax test 2.13% and SNIP test 1.86% (not shown in figures separately), contributes 10.52%, and is the second most important factor in prediction. Respiratory function parameters are the most significant, among the top 10 factors, in predicting FEV1% on the fourth postop day, as shown in Fig. 3b.

On the seventh postoperative day, the effect of diaphragm mobility is 5.63%, PImax 1.17%, and SNIP 2.26% (not shown in figures separately) which makes the total contribution of the diaphragm 9.06% and is the third most important factor in prediction. The spirometry parameters lead in importance; see Fig. 3c.

## Discussion

PpoFEV1% is a parameter that is widely used in postoperative mortality and morbidity assessment and is recommended according to ERS/ESTS guidelines [1]. The loss of pulmonary function is the greatest in the first postoperative week and improves afterwards in the following 6 months when it stabilizes at a lower level than it was preoperatively. After surgery, reduced respiratory function is not only the result of lung volume loss but also impairment in chest wall compliance and lung compliance which is related to the accumulated bronchial secretion, bronchial hyperreactivity, microatelectasis, increased lung water, and reduced surfactant activity. Also, the diaphragm function is eroded. The aforementioned impairments gradually improve after surgery with time [24].

It is presumed that the respiratory function recovery within 30 days of surgery is also a consequence of chest wall surgical injury healing and alleviation of surgical pain [6]. It is previously shown that the lower the pain, the higher the postoperative FEV1% [7]. The machine learning model in our study suggests that surgical pain had no influence on postoperative measured FEV1%.

Almost every potential cardio and respiratory complication occurs immediately after surgery [25–27], and therefore, predicting FEV1% in the immediate postoperative period is important because it is shown that the measured FEV1% on the first postoperative day is more significant in assessing risks of postoperative complications than ppoFEV1% [27].

The measured FEV1% in our patients immediately after surgery correspond to the values published in earlier literature, and their values recover from the first to the seventh postoperative day, and they differ from the ppoFEV1% obtained by segment counting methods [7, 25].

Segment counting methods for predicting FEV1% overestimate the measured FEV1% in the first postoperative days [7], which was noted in our study as well.

Machine learning models have been used in clinical studies to estimate outcomes, predict postoperative lung function and risk of complications after lung surgery, and are shown to be more precise than standard statistical methods [12–14]. The machine learning model that was applied in this study predicts the postoperative FEV1% significantly better (two to three times, measured by MAE) in the immediate postoperative period in comparison to segment counting methods that are widely used in clinical practice [6]. The proposed regression models are specifically trained to predict FEV1% for the 1st, 4th, and 7th day after surgery and were proven to be highly accurate (with a mean absolute error in the range between 8 and 11%).

Meta-analysis that included 17 studies showed that prediction of FEV1% after lung surgery is more precise when computed tomography volume and density measures were combined. However, in every study included in this analysis, respiratory function prediction is performed for the period at least 3 months after surgery, and it is concluded that every available method is imprecise [28].

Methods of postoperative prediction of respiratory function do not take into account the impact of the diaphragm. Several meta-analyses indicated that preoperative respiratory muscle training could contribute to larger respiratory muscle strength postoperatively, and that would significantly reduce the occurrence of respiratory complications. This benefit is especially observed in older patients, higher risk patients, and thoracic surgery patients [9, 29, 30]. The aforementioned findings indicate the potential significance of inspiratory muscles, the diaphragm primarily, in postoperative prediction of respiratory function.

We demonstrated that the diaphragm plays a big role in postoperative respiratory function prediction in lung cancer surgical patients.

We used noninvasive methods easily available in clinical practice in assessment of the diaphragm function.

Diaphragm function is presented in our study by diaphragm mobility measured both by ultrasound and chest radiography and noninvasive muscle strength tests. Considering that ultrasound and chest radiography capture different aspects of diaphragm mobility, ultrasound measures mobility of its posterior parts, while chest radiography measures mobility of its dome, anterior parts, and diaphragm mobility is represented as the sum of these values. Our machine learning model allowed us to do that by recognizing them as different independent variables in output prediction. It has been shown previously by ultrasound that diaphragm mobility is greater posteriorly than anteriorly and greater laterally than medially which was confirmed by dynamic magnetic resonance imaging at deep breathing [31, 32].

Considering that inspiratory muscle strength tests, PImax, and especially SNIP present diaphragm muscle strength to the largest extent, their values combined with diaphragm mobility represent diaphragm function as a single appropriate parameter [16].

Our study shows that the diaphragm has a great role in the prediction of respiratory function in the immediate postoperative period in lung cancer surgical patients. The diaphragm function impact gradually decreases from the first to the seventh day, while at the same time, the impact of preoperative respiratory function increases, which could be a consequence of chest wall and lung compliance recovery [24].

This prediction model of respiratory function has advantages because it is equally applicable in prediction after lobectomy and pneumonectomy, while both the Nakahara and functional segment methods are not suitable in prediction after pneumonectomy [8].

Our prospective study has several limitations. The main limiting factor in the design of the regression metamodel is the relatively small training set. With its increase, the possibility of more precise adjusting hyperparameters of the base models opens up, which further improves the accuracy of the entire model.

In the analysis, diffusing lung capacity for carbon monoxide (DLCO) was not taken into account. It is recommended to be used in current preoperative patient assessment according to guidelines [1]. Future studies are to include it.

The vast majority of patients in our study had normal respiratory function or mild COPD. Since the study had only one patient with severe COPD, future studies are called for to estimate the results in patients with moderate to severe COPD who need studies like this the most.

## Conclusion

The obtained results indicate that the accuracy of the prediction using the metamodel is significantly higher compared to traditional calculations. This is one of the rare analyses that study the effect of the diaphragm on postoperative respiratory function prediction in lung cancer surgical patients. Our analysis unequivocally established a correlation between diaphragm and respiratory function. Using our model and its results, it can be concluded that the diaphragm cannot be ignored and plays a notable part in FEV1% prediction in the immediate postoperative period.

A special contribution of our model is that its improved prediction of respiratory function could contribute to better quality of surgical patient selection.

## Data Availability

The datasets used and/or analyzed in the current study are available through the corresponding author on reasonable request.
